# The Teeth in Literature

**Published:** 1876-03

**Authors:** W. C. Barrett


### DENTAL REGISTER.

VOL. XXX.] MARCH, 1876. [No. 3.

### THE TEETH IN LITERATURE.

BY W. C. BARRETT, M. D. S.

From the earliest times, attention has been paid to the teeth, not only as important organs in the human mechanism, but as affording indications extremely useful in the diagnosis, or prognosis of disease. Among physicians of to-day, an examination of the oral cavity is almost always preliminary to an opinion; but the inspection usually goes no further than the tongue, which indicates but the then existing condition of the patient; whereas the teeth often bear marks, which to an intelligent observer, reveal all the previous sanitary history.

Hippocrates, the Father of Medicine, in his Prognostics and Prophetics, frequently alludes to the importance of a close attention to them, as indicating the progress of morbific tendencies. Indeed, he has many wise suggestions, that it would be well if the dental, as well as medical profession of this age, would ponder and study over.

In his Aphorisms, (18) he says: "Cold is inimical to the bones, the teeth, the nerves, the brain, and the spinal marrow." This, Theophilus and Galen explain, as meaning that the parts of the body here mentioned, are of a cold nature; possessed of little vascularity, and hence readily injured by a low temperature. It is an acknowledged fact, that the frequent neuralgias, and rheumatisms of the parts enumerated, may often be referable to thermal changes.

The Bible has frequent reference to the teeth, and their relative importance in the human economy. In the Mosaic law, which was a code of retaliation and reprisals, special provision was made for the punishment of those, through whose crime or fault, another might lose a tooth. In the twenty-first chapter of Exodus, the general common law is laid down: "An eye for an eye, and a tooth for a tooth." Afterwards, in legislating upon the relation of master and servant, the great lawgiver says concerning this matter: "If he smite out his man-servant's tooth, he shall let him (the servant) go free, for his tooth's sake." In those times, the opinion was not entertained, that the teeth were furnished men for the express purpose of being removed for the insertion of artificial ones. It was not till this modern period of "cheap and nasty" dentistry, that such a thing was thought of. And yet, in those early days, some nations at least, were not without dentists and dentistry. Inspection of the remains found in some ancient tombs, show conclusively, that they were conversant with mothods of filling carious teeth, and even of inserting an artificial substitute, in cases of unavoidable mutilation.

Among the ancient Egyptians, dentists were common, though they formed a part of the medical profession. Herodotus says:—(Enterpe Cap. 84,) "The art of medicine is thus divided among them: each physician applies himself to one disease only, and not more. All places abound in physicians; some physicians are for the eyes, others for the head,

*others for the teeth*, others for parts about the belly, and others for internal disorders."

This author also gives an account of one of the most interesting dental anomalies, of which there is any mention. He says:—(Calliope Cap. 83.) "After the battle of Platea, and when the Plateans brought the bones together in one place, there was discovered a jaw, and the upper jaw had teeth growing in a piece, all in one bone, both the front teeth and the grinders."

In all ages, the possession of a full and perfect set of dentures has been highly prized. Hear the opinion of the great satirist, Cerantes Don Quixote, after his famous rencontre with the shepherds, calls upon his squire to examine into the havoc made among his teeth, by the stones slung by his adversaries. "Pray sir!" said Sancho, "how many grinders did your worship use to have upon that side?" "Four," answered the Don, "if not five; besides the eyetooth; for I never in all my life have had a tooth drawn, or dropped out, or *rotted out by the worm*, or loosened by rheum," "Bless me!" quoth Sancho Panza; "why you have in this nether jaw, on this side but two grinders and a stump, and in that part of your upper jaw, never a stump and never a grinder. Alas! all is leveled there as smooth as the palm of one's hand." "Unfortunate that I am," said the Knight. "I had rather have lost an arm, so it were not my sword arm; for a mouth without cheek teeth, is like a mill without a stone, Sancho; and every tooth in a man's head, is more valuable than a diamond."

The teeth are frequently introduced as the foundation of some rhetorical figure. Montaigue uses them thus, to illustrate the same utter destruction which is spoken of in the Bible, as coming upon that which is crushed between "the upper and nether millstones." Indeed the Bible itself is full of such passages. The prophet Amos uses the term—"cleanness of teeth," as a synonym for starvation. Ezekiel, and Jeremiah, to indicate in a periphrastic manner, the hereditary consequence of crime, say: "The parents have eaten sour grapes, and the children's teeth are set on edge." In Solomon's song, the picture of the perfect woman is not complete without the verse—"Thy teeth are as a flock of sheep that go up from the washing, whereof every one beareth twins; and there is not one barren among them"—referring to the appearance in pairs, of the teeth of the perfect dentition. Job says of his great peril—"I am escaped by the skin of my teeth." The torments of the last, are indicated by St. Mathew, in the expression—"there shall be gnashing of teeth." Rabelais, however, paints the lily by representing that in the hereafter, dentists shall figure among the tormentors; for he says that in the world of evil spirits—"the four sons of Aymon are tooth drawers." Thorean, the poet-naturalist, uses modern dentistry in a more complimentary simile; for he says—"The most of our expected ills are like the dentist's chair; much worse in the anticipation than in the actual amount of suffering experienced."

The importance of the teeth has always given birth to many aphorisms and proverbs in all languages. Of a fully equipped soldier we say:—"he is armed to the teeth." Of a pugnacious individual: "he fights tooth and toe nail;" of a disputant: it was "proved to his teeth." A man is taunted by having a disagreeable fact "thrown in his teeth." One who, by adverse experience gains knowledge, is said to "cut his eye teeth."

The disease of teeth are often referred to in literature, as amongst the most intolerable of the minor ills, to which flesh is heir. Shakespeare says "I never yet knew a philosopher who could endure the tooth ache patiently." Byron speaks of tooth ache, as the "Hell of all diseases." Dr. Johnson, the "Great Cham of Literature," said; "Laziness is worse than the tooth ache;" thereby intimating that as the latter is the most unbearable of the bodily ills, so is the former the worst of moral diseases.

One of the oldest of Mother Goose's melodies, (and few are aware of the antiquity of some of them) refers to a juvenile patient and his cure.

"Little Tommy Grace had such a pain in his face,
So bad he could not say a letter;

When in came Sicky Long, singing such a funny song,
That Tommy laughed and found his face much better."

Vishna Sarma, that rare book of the Sanscrit, whose authorship is lost in the mists of antiquity, the teeth and their diseases are frequently used to point a moral. Here is one of the apothegms of that books.

"It is better to tear up by the roots a rotten tooth, a faithless servant, and a wicked minister."

Small as these organs are, their relative importance has not, in literature, been over rated. The whole process of nutrition is, to a large degree, dependent upon them. Situated as they are in the gateway of the alimentary canal, they meet the food at its entrance into the system, and their office, upon which the other functions are dependent, is the first to be performed. If the aliment be not properly prepared by mastication, it is useless for any purpose of nourishment. Mankind has had no need to be told this by the dentist, for there are few things, so thoroughly accepted in literatnre, as these truths, the practical application of which men so frequently ignore.

